# Hydrocortisone for Preventing Adverse Drug Reactions to Snake Antivenom: A Meta-Analysis

**DOI:** 10.1155/2022/6151206

**Published:** 2022-04-22

**Authors:** Jihua Feng, Zimeng Wu, Qiao Yu, Hongyuan Li, Pan Ji, Yanli Yang, Xiaoliang Zeng, Xiaowen Zheng, Chunling Zhao, Jianfeng Zhang

**Affiliations:** Department of Emergency Medicine, The Second Affiliated Hospital of Guangxi Medical University, Nanning 530007, China

## Abstract

**Objective:**

Pretreatment with hydrocortisone (prehydrocortisone) has been used to protect against adverse drug reactions (ADRs) following antivenom administration after snakebite. However, controversial results have been reported in studies evaluating its efficacy. Herein, we conducted a meta-analysis to evaluate the effect of prehydrocortisone on the risk of ADRs.

**Methods:**

We conducted a systematic search of PubMed, Embase, and Cochrane for relevant studies on the literature published up to December 6, 2020, with no language restrictions. Premedications, including hydrocortisone with or without other drugs, were compared with placebo or no premedication. Our primary end point was the risk of ADRs, which was reported as the number of patients who developed ADRs divided by the total number of snakebite patients administered with antivenom separately for the prehydrocortisone and control groups for each study. We evaluated pooled data using of a random-effects model.

**Results:**

Among 831 identified studies, 4 were eligible and included in our analysis (*N* = 1348 participants). Upon combining all eight comparisons from the four selected studies, the overall pooled odds ratio (OR) for ADRs was 0.47 (95% CI 0.19, 1.17; *p*=0.11; *I*^*2*^ = 68%). When the analysis was restricted to only articles using hydrocortisone with other drugs, the pooled OR was 0.19 (95% CI 0.05, 0.75; *p*=0.02; *I*^*2*^ = 55%). The result was not statistically significant when the analysis was restricted to studies using prehydrocortisone alone, or randomized controlled designs, or cohorts. Our study was limited by heterogeneity, quality, and a paucity of data.

**Conclusions:**

The findings in this study revealed that prehydrocortisone alone was ineffective. However, the substantial beneficial effect of prehydrocortisone combinations with premedications (injectable antihistamines or adrenaline) used against ADRs cannot be excluded. Therefore, the use of prehydrocortisone combinations with premedications (injectable antihistamines or adrenaline) as a prophylaxis may reduce the ADRs to antivenom.

## 1. Introduction

Snakebite is an important public health issue in many tropical and subtropical agricultural communities in developing countries [1]. Antivenom has been used to manage snakebites since its development in 1898 by Albert Calmette in Vietnam [2], and effective antivenom remains the only specific therapy for snakebite envenomation. However, in addition to the desired therapeutic effects, antivenoms result in adverse drug reactions (ADRs), including life-threatening anaphylaxis and cardiorespiratory emergences [3]. Unfortunately, ADRs are unreliable and not predictive based on sensitivity tests for the occurrence of early reactions and severe systemic anaphylaxis [4]. Acute ADR rates (ranging within 30%–81%) after antivenom administration are commonly reported in certain parts of the tropics [5–8]. Among them, life-threatening ADRs ranged from 13% to 35% [5, 6, 9, 10].

In areas, where snakebite is common, qualified workers and equipment are often lacking in health facilities. It is desirable to take actions to safely reduce the risk of ADRs of antivenom. A safe and efficacious premedication to prevent potentially life-threatening anaphylaxis is important in the management of snakebites in these regions.

Premedications, such as hydrocortisone, adrenaline, and antihistamine, have been used to prevent ADRs following antivenom treatments with variable results [3, 6, 7, 11–13]. A randomized controlled trial (RCT) has demonstrated the effectiveness of intravenous hydrocortisone infusion in preventing ADRs if administered simultaneously with the antivenom infusion together with chlorpheniramine [6]. Conversely, in another clinical trial, intravenous hydrocortisone alone administered at the starting point of the antivenom infusion did not significantly reduce the ADRs versus placebo [5]. In 2011, a meta-analytical review reported that prophylactic adrenaline alone was beneficial, and it was marginally beneficial in combination with other premedications (e.g., injectable hydrocortisone and antihistamines) in the reduction of ADRs [14]. In consideration of these developments and subsequent published articles, in the present study, we conducted a meta-analysis addressing the question of whether precaution with hydrocortisone (perhydrocortisone) prevents ADRs following antivenom administration.

## 2. Methods

### 2.1. Search Strategy

This systematic review and meta-analysis is reported in accordance with the Preferred Reporting Items for Systematic Reviews and Meta-Analyses (PRISMA) statement. However, the study protocol was not registered.

We selected relevant studies on published literature up to December 6, 2020, by searching PubMed, Embase, and Cochrane. We applied no language restrictions with no period or regional restrictions. We used the following combined text and MeSH terms: “Snake Bites,” “Antivenom,” “Anaphylaxis” “Hydrocortisone,” and “Glucocorticoids.” Appendix 1 provides details of the PubMed search strategy. We considered all potentially eligible studies for review irrespective of the primary outcome or language. We also performed a manual search using the reference lists of key published articles.

### 2.2. Selection Criteria

We reviewed the full text of all accessible studies evaluating or reporting the prevention of ADRs following antivenom use after snakebite. We regarded studies as eligible for inclusion if they were RCTs or cohort studies (prospective cohorts, retrospective cohorts, and historical cohorts) conducted in snakebite victims who received antivenoms and prehydrocortisone versus controls (defined as placebo or no premedication). The risks of ADRs were then compared. The exclusion criteria were as follows: studies that did not fulfill the inclusion criteria, had insufficient information, and/or lacked desired information. The Cochrane Risk of Bias 2.0 (ROB-2) tool and Newcastle-Ottawa Scale (NOS) scores were further calculated to estimate the quality and validity of the studies. We assessed the evidence for each outcome using the Grading of Recommendations, Assessment, Development, and Evaluation (GRADE) approach.

### 2.3. Data Extraction

We extracted the following data from each selected study: study participants, number of participants with ADRs, design, antivenoms administered, premedication intervention-drug regimen, and other outcomes recorded.

Two independent investigators (JHF and ZMW) reviewed the study titles and abstracts, and studies that satisfied the inclusion criteria were retrieved for full-text assessment. Trials selected for detailed analysis and data extraction were analyzed by two investigators (JHF and ZMW) with an agreement value (*κ*) of 96.5%; disagreements were resolved by a third investigator (JFZ).

### 2.4. Statistical Analysis

We compared prehydrocortisone with or without other drugs with placebo or no premedication. The outcomes assessed were as follows: risk of ADRs as the number of patients who developed ADRs divided by the total number of snakebite patients administered antivenom separately for the prehydrocortisone and control groups for each study.

We calculated pooled estimates of the odds ratio (OR) between prehydrocortisone groups using a random-effects model (REM) of the effect of prehydrocortisone. Individual studies with data that could be categorized into prehydrocortisone alone, prehydrocortisone with other drugs, or other mutually exclusive premedication groups were analyzed as separate substudies or comparisons as prespecified. In this meta-analysis, all studies were first considered as a single group. Subsequently, four separate meta-analyses were performed with studies restricted to only RCTs, nonrandomized cohort, or comparative studies, studies containing prehydrocortisone alone and studies containing prehydrocortisone with other drug premedications.

Preplanned sensitivity analyses were restricted to investigate the influence of a single study by reducing the heterogeneity of the treatment-induced changes in outcomes in the comparator arm seen in the overall analysis.

We investigated the possibility of publication bias by constructing a funnel plot of each trial's effect size against the standard error. We assessed funnel plot asymmetry using Begg and Egger tests and defined significant publication bias as a *p* value <0.1. Given the limitations of the funnel plot, publication bias was only confirmed when detected in both tests used for its assessment.

We used the Cochran *Q* test to assess heterogeneity between studies. *I*^*2*^ testing was used to assess the magnitude of the heterogeneity between studies with values greater than 50% regarded as indicating moderate-to-high heterogeneity. We used Review Manager (version 5.4) and Stata (version 11.0) for all statistical analyses.

## 3. Results

**Figure 1:**
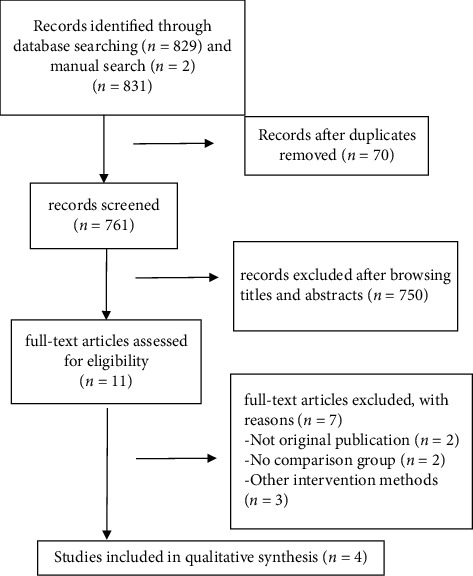
Study selection process.

We identified 831 articles, of which 4 (with data for 1348 participants) were included in our analysis (Figure 1). All four studies were published between 2004 and 2011 (Table 1). Two of the studies were RCTs (low ROB for two RCTs) and two were retrospective cohorts (NOS score of 5 for the two cohorts).  Table 2 presents the pooled outcomes with associated GRADE certainty of evidence.

Data extracted from patients in two of the four studies could be categorized into recipients of prehydrocortisone alone or prehydrocortisone with other drug groups. Each study was analyzed as a separate comparison or a substudy identified as (a), (b), (c), and (d) based on their respective interventions.

**Figure 2:**
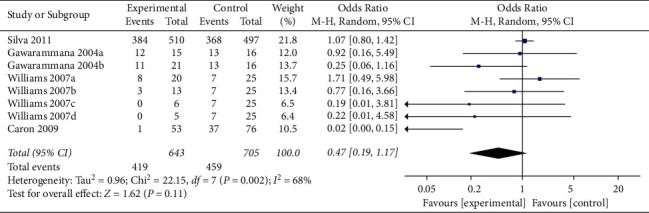
Forest plot of ADRs associated with antivenom use following snakebite between the prehydrocortisone with or without other drugs groups and the placebo or no premedication groups in eight comparisons from four studies.

**Figure 3:**
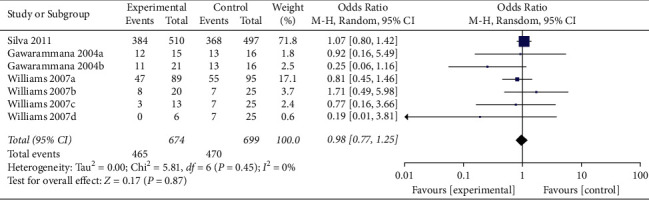
Sensitivity analysis of ADRs associated with antivenom use following snakebite between the prehydrocortisone with or without other drugs groups and the placebo or no premedication groups in seven comparisons from three studies.

In a pooled analysis of all four studies, the OR of ADRs was 0.47 (95% CI 0.19, 1.17; *p*=0.11; *I*^*2*^ = 68%) with statistically significant between-study heterogeneity (Figure 2). In six of eight comparisons, the point estimate of OR was <1. The funnel plot (Appendix 2) suggested that no publication bias was evident (*p*=0.174 and *p*=0.121 using Begg's and Egger's tests, respectively). Sensitivity analysis was performed by excluding each article from the summary results to distinguish the source of heterogeneity and evaluate the robustness of the results. Sensitivity analysis showed that the OR for seven comparisons from three studies combined was 0.98 (95% CI 0.77, 1.25; *p*=0.87; *I*^*2*^ = 0%) with no significant between-study heterogeneity (Figure 3).

**Figure 4:**
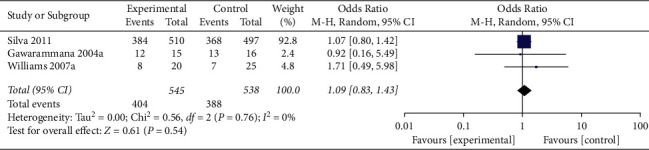
Forest plot of ADRs associated with antivenom use following snakebite between the prehydrocortisone alone groups and the placebo or no premedication groups in three comparisons from three studies.

Restricting analysis to studies using prehydrocortisone alone revealed that the summary OR for three comparisons from the three studies combined was 1.09 (95% CI 0.83, 1.43; *p*=0.54; *I*^*2*^ = 0%) with no significant between-study heterogeneity (Figure 4). The funnel plot suggested that ublication bias was evident (*p*=0.296 and *p*=0.567 using Begg's and Egger's tests, respectively).

**Figure 5:**
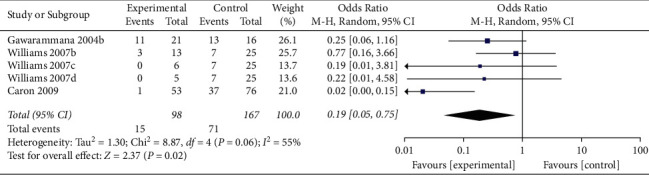
Forest plot of ADRs associated with antivenom use following snakebite between the prehydrocortisone with other drugs groups and the placebo or no premedication groups in five comparisons from three studies.

**Figure 6:**
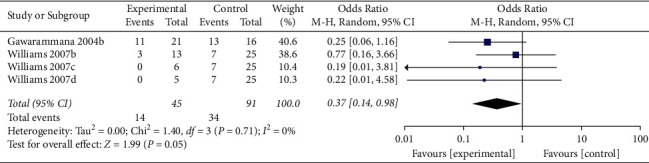
Sensitivity analysis of ADRs associated with antivenom use following snakebite between the prehydrocortisone with other drugs groups and placebo or no premedication groups in four comparisons from two studies.

When the analysis was restricted to studies using prehydrocortisone with other drugs, the summary OR for five comparisons from three studies combined was 0.19 (95% CI 0.05, 0.75; *p*=0.02; *I*^*2*^ = 55%) with moderate between-study heterogeneity (Figure 5). The funnel plot suggested that no publication bias was evident (*p*=0.462 and *p*=0.364 using Begg's and Egger's tests, respectively). Excluding individual studies yielded similar overlapping estimates. Sensitivity analysis showed that the OR for four comparisons from two studies combined was 0.37 (95% CI 0.14, 0.98; *p*=0.05; *I*^2^ = 0%) with no significant between-study heterogeneity (Figure 6).

**Figure 7:**
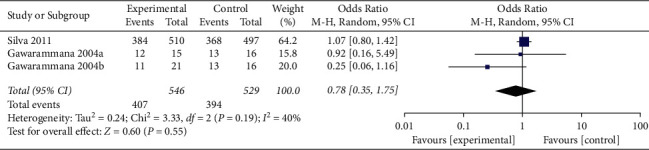
Forest plot of ADRs associated with antivenom use following snakebite between the prehydrocortisone with or without other drugs groups and the placebo or no premedication groups in three comparisons from two RCTs.

When the analysis was exclusively restricted to RCTs the summary OR for three comparisons from two studies combined was 0.78 (95% CI 0.35, 1.75; *p*=0.55; *I*^*2*^ = 40%) with mild between-study heterogeneity (Figure 7). The funnel plot suggested that no publication bias was evident (*p*=1.00 and *p*=0.468 using Begg's and Egger's tests, respectively).

**Figure 8:**
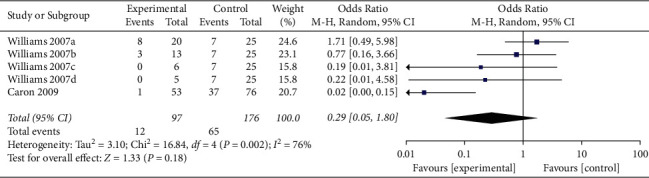
Forest plot of ADRs associated with antivenom use following snakebite between the prehydrocortisone with or without other drugs groups and the placebo or no premedication groups in five comparisons from two cohort studies.

**Figure 9:**
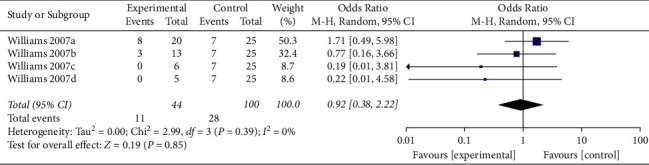
Sensitivity analysis of ADRs associated with antivenom use following snakebite between the prehydrocortisone with or without other drugs groups and the placebo or no premedication groups in four comparisons from one cohort study.

When the analysis was exclusively restricted to cohort studies, the summary OR for five comparisons from two studies combined was 0.29 (95% CI 0.05, 1.80; *p*=0.18; *I*^*2*^ = 76%) with statistically significant between-study heterogeneity (Figure 8). The funnel plot suggested that no publication bias was evident (*p*=0.462 and *p*=0.251 using Begg's and Egger's tests, respectively). Excluding individual studies yielded similar overlapping estimates. Sensitivity analysis showed that the combined OR for four comparisons from one study was 0.92 (95% CI 0.38, 2.22; *p*=0.85; *I*^*2*^ = 0%) with no significant between-study heterogeneity (Figure 9).

## 4. Discussion

The combined summary OR for prehydrocortisone with antihistamines or adrenaline premedication was statistically significant; however, our study highlighted the limitations and paucity of existing data. Restricting the analysis to cohorts or RCTs shows a lack of beneficial effects using appropriate statistical methods.

Hydrocortisone together with antihistamines or adrenaline conferred approximately 81% protection against ADRs, and the benefit was reported in three of the five comparisons [6, 15, 16]. Premawardhena et al. reported that the group receiving adrenaline (adrenaline against a saline control) suffered fewer ADRs overall [7]. Fan et al. found that the group receiving antihistamineexhibited no differences in ADR compared with the group receiving placebo [11].

The overall summary OR for ADRs after treatment with prehydrocortisone combinations was not significant, although the point estimate was 0.47, with boundaries of the 95% CI 0.19 and 1.17. The combined summary OR for hydrocortisone premedication alone was not significant, and the point estimate and boundaries of the 95% CI suggested that studies using hydrocortisone premedications alone failed to show any beneficial effects. A lack of effectiveness was reported in three of the three comparisons. This ineffectiveness was more pronounced than that reported in a previous systematic review and meta-analysis, which was based on fewer studies with hydrocortisone-containing premedication [14]. Moreover, in the current study, corticosteroids take longer to act, but Kularatne et al. found that hydrocortisone neither reduces the occurrence nor delays ADR associated with antivenom serum during the first hour and the first 48 h when hydrocortisone prophylaxis was administered 2 h and up to 4 h prior to antivenom serum administration [12].

Acute allergic reaction is a predictable toxicity of antivenom serum. Antivenoms commonly used in South Asian countries were associated with an incidence of severe ADRs as high as 20 to 40% [10, 17]. Variawa et al. reported that 57% of patients who received antivenom suffered from acute anaphylactic reactions in South Africa [18]. In addition, recurrent ADRs may relapse acutely when additional doses of antivenom are administered as infusions or intravenous pushes and “accidentally” as biphasic anaphylaxis hours after the prodromal symptoms disappear obviously [19]. In addition, despite the initial good clinical response to antivenom serum, patients may experience “accidentally” worsed clinical symptoms because their venom relapses. Sanjib et al. reported that acute severe and recurrent antivenom-related anaphylaxis was common and recurrent and exhibited overlapping signs with severe neurotoxic envenomation [9]. The main ADRs of the four studies included in our analysis were allergies [5, 6, 15, 16].

The mechanisms of action of glucocorticoids are diverse and may persist for a long time. These multiple mechanisms include immunosuppressive properties, antiinflammatory activity, and antiproliferative effects. Immunosuppressive properties reduce the response to type III and type IV hypersensitivity reactions. Glucocorticoids are known as inhibitors of mast cell degranulation. Glucocorticoids have been widely used to treat many pathological processes related to mast cell degranulation, including allergic reactions. Stone et al. provided a large amount of evidence that the allergic reaction caused by antivenom treatment is mainly due to mast cell degranulation rather than complement activation, as previously mentioned [20]. Mast cell degranulation following antivenom treatment is suspected to be related to possible IgG microaggregates in antivenom and may be related to the priming effect of venom-induced complement activation [20]. In our study, prehydrocortisone treatment with antihistamines or adrenaline had an OR of 0.19 (95% CI 0.05, 0.75), suggesting that hydrocortisone may play a role in reducing the incidence of ADRs caused by antivenom treatment. However, when using prehydrocortisone alone, the OR was 1.09 (95% CI 0.83, 1.43), suggesting that prehydrocortisone alone was ineffective. However, our analysis is insufficient based on the available data from only two RCTs and two cohorts; the study findings should be interpreted with caution.

Publication bias is less likely to have affected the results of this analysis given that most of the articles did not report positive results, which compared with negative articles, are more likely to be published. Moreover, using multiple approaches, these analyses implied that publication bias did not affect the pooled estimates. However, the level of publication bias due to the lack of literature inclusion is unclear but is likely to be minimal.

A limitation of this analysis is the insufficiency of existing data as only two RCTs and two cohorts were available for inclusion in this study. Second, the articles have some sources of heterogeneity, including differing definitions of reactions severity, manifestations of envenomation among patients, study designs, and premedications. This heterogeneity provides some advantages from the view of universality of findings to various patient populations [21, 22]. Furthermore, we performed restricted subanalyses on the data obtained from randomized and nonrandomized research using hydrocortisone with (without) antihistamines or adrenaline premedication separately to address these challenges. Third, ADRs were perhaps more common in victims with lower levels of venom proteins in the blood. Additionally, some differences in ADR rates may have depended on the species of envenoming snake and on the degree of envenomation. In this article, these issues were not resolved. Fourth, it is also important to recognize that research from Papua New Guinea and Sri Lanka involved high-quality antivenom, and the most of adverse reactions were generally pyrogenic or cutaneous. These results suggest that these reactions were less severe than adverse events observed in other settings, where relatively low-quality antivenoms that result in a high proportion of more severe adverse reactions (including anaphylaxis) are used. Hence, the effect of premedication in reducing ADRs may have become much less dramatic in the settings using refined antivenoms. Caron et al. reported that the slow infusion of antivenom and premedication over 1 h compared with an intravenous push injection reduced both the frequency and severity of ADRs [16]. However, Isbister et al. reported that the infusion of antivenom at a slow rate did not reduce the severe ADRs [23]. These issues were not solved in our study. Finally, the timing for administering premedications before antivenom administration differs. Other limitations included poor reporting of ADRs, small sample sizes for most articles, varying methodological quality of research, and study protocols that were not registered. A detailed account of such variant methodological and reporting discrepancies in clinical research on snakebites was published in 2010 [24].

## 5. Conclusions

The findings in this study revealed that prehydrocortisone alone was ineffective. However, the substantial beneficial effect of prehydrocortisone combinations with premedications (injectable antihistamines or adrenaline) used against ADRs cannot be excluded. Therefore, the use of prehydrocortisone combinations with premedications (injectable antihistamines or adrenaline) as a prophylaxis may reduce the ADRs to antivenom.

## Figures and Tables

**Table 1:** Characteristics of included studies.

Study, y	Design	Interventions	No reactions/total (%) in two comparative arms	OR (95% CI)	Potential risk for bias
de Silva et al., 2011 [5]	Randomized, double- blind, PL-controlled	Hydrocortisone IV vs PL	384/510 (76.3) vs 368/497 (74.0)	1.07 (0.80, 1.42)	Low risk of confounding because of randomized design
Gawarammana et al., 2004a [6]	Randomized, double- blind, PL-controlled	Hydrocortisone IV vs PL	12/15 (92.3) vs 13/16 (81.3)	0.92 (0.16, 5.49)	Premature trial stoppage. Low statistical power from small sample size
Gawarammana et al., 2004b [6]	Randomized, double- blind, PL-controlled	Hydrocortisone + chlorpheniramine vs PL	11/21 (52.4) vs 13/16 (81.3)	0.25 (0.06, 1.16)	Premature trial stoppage. Low statistical power from small sample size
Williams et al., 2007a [15]	Retrospective cohort	Hydrocortisone IV vs no premedication	8/20 (40.0) vs 7/25 (28.0)	1.71 (0.49, 5.98)	Potential confounding—hydrocortisone with other drug group also received other agents. ADR may have been missed as nonurticarial type often unrecognized. Low quality of medical records in rural areas
Williams et al., 2007b [15]	Retrospective cohort	Hydrocortisone + promethazine vs no premedication	3/13 (23.1) vs 7/25 (28.0)	0.77 (0.16, 3.66)	ADR may have been missed as nonurticarial type often unrecognized. Low quality of medical records in rural areas
Williams et al., 2007c [15]	Retrospective cohort	Hydrocortisone + adrenaline vs no premedication	0/6 (0) vs 7/25 (28.0)	0.19 (0.01, 3.81)	ADR may have been missed as nonurticarial type often unrecognized. Low quality of medical records in rural areas
Williams et al., 2007d [15]	Retrospective cohort	Hydrocortisone + adrenaline + promethazine vs no premedication	0/5 (0) vs 7/25 (28.0)	0.22 (0.01, 4.58)	ADR may have been missed as nonurticarial type often unrecognized. Low quality of medical records in rural areas
Caron et al., 2009 [16]	Historical cohort	Hydrocortisone + diphenhydramine vs no premedication	1/53 (1.9) vs 37/76 (48.7)	0.02 (0.00, 0.15)	The two groups not strictly similar. Control group evaluated retrospectively and intervention group prospectively. Different antivenom administration methods

**Table 2:** The GRADE approach to evaluate the quality of evidence.

Hydrocortisone for preventing adverse drug reactions to snake antivenom: a meta-analysis						
Patient or population: patients with antivenom use after snakebite Intervention: the prevention of ADRs following antivenom use after snakebite						

Outcomes	Illustrative comparative risks^*∗*^ (95% CI)		Relative effect (95% CI)	No of participants (studies)	Quality of the evidence (GRADE)	Comments
	Assumed risk	Corresponding risk				
	Control	Experimental				

The ADRs associated with antivenom use following snakebite between the prehydrocortisone with or without other drugs groups and the placebo or no premedication groups in eight comparisons from four studies.	Study population		OR 0.47 (0.19 to 1.17)	1348 (8 studies)	⊕⊕⊕⊝ low^1^	
	651 per 1000	467 per 1000 (262 to 686)				
	Moderate					
	383 per 1000	226 per 1000 (105 to 421)				

The ADRs associated with antivenom use following snakebite between the prehydrocortisone with or without other drugs groups and the placebo or no premedication groups in three comparisons from two RCTs	Study population		OR 0.78 (0.35 to 1.75)	1075 (3 studies)	⊕⊕⊕⊝ low^2^	
	745 per 1000	695 per 1000 (505 to 836)				
	Moderate					
	813 per 1000	772 per 1000 (603 to 884)				

The ADRs associated with antivenom use following snakebite between the prehydrocortisone with or without other drugs groups and the placebo or no premedication groups in five comparisons from two cohort studies	Study population		OR 0.29 (0.05 to 1.8)	273 (5 studies)	⊕⊕⊕⊝ very low^2^	
	369 per 1000	145 per 1000 (28 to 513)				
	Moderate					
	280 per 1000	101 per 1000 (19 to 412)				

Sensitivity analysis of ADRs associated with antivenom use following snakebite between the prehydrocortisone with or without other drugs groups and the placebo or no premedication groups in seven comparisons from three studies.	Study population		OR 0.98 (0.77 to 1.25)	1373 (7 studies)	⊕⊕⊕⊝ low^3^	
	672 per 1000	668 per 1000 (612 to 720)				
	Moderate					
	579 per 1000	574 per 1000 (514 to 632)				

Sensitivity analysis of ADRs associated with antivenom use following snakebite between the prehydrocortisone with or without other drugs groups and the placebo or no premedication groups in four comparisons from one cohort study.	Study population		OR 0.92 (0.38 to 2.22)	144 (4 studies)	⊕⊕⊕⊝ very low^2^	
	280 per 1000	264 per 1000 (129 to 463)				
	Moderate					
	280 per 1000	264 per 1000 (129 to 463)				

The ADRs associated with antivenom use following snakebite between the prehydrocortisone alone groups and the placebo or no premedication groups in three comparisons from three studies.	Study population		OR 1.09 (0.83 to 1.43)	1083 (3 studies)	⊕⊕⊕⊝ low^4^	
	721 per 1000	738 per 1000 (682 to 787)				
	Moderate					
	740 per 1000	756 per 1000 (703 to 803)				

The ADRs associated with antivenom use following snakebite between the prehydrocortisone with other drugs groups and the placebo or no premedication groups in five comparisons from three studies.	Study population		OR 0.19 (0.05 to 0.75)	265 (5 studies)	⊕⊕⊝⊝ low	
	425 per 1000	123 per 1000 (36 to 357)				
	Moderate					
	280 per 1000	69 per 1000 (19 to 226)				

Sensitivity analysis of ADRs associated with antivenom use following snakebite between the prehydrocortisone with other drugs groups and placebo or no premedication groups in four comparisons from two studies.	Study population		OR 0.37 (0.14 to 0.98)	136 (4 studies)	⊕⊕⊝⊝ low	
	374 per 1000	181 per 1000 (77 to 369)				
	Moderate					
	280 per 1000	126 per 1000 (52 to 276)				

^*∗*^The basis for the assumed risk (e.g., the median control group risk across studies) is provided in footnotes. The corresponding risk (and its 95% confidence interval) is based on the assumed risk in the comparison group and the relative effect of the intervention (and its 95% CI). CI: confidence interval; OR: odds ratio; GRADE working group grades of evidence. High quality: further research is very unlikely to change our confidence in the estimate of effect. Moderate quality: further research is likely to have an important impact on our confidence in the estimate of effect and may change the estimate. Low quality: further research is very likely to have an important impact on our confidence in the estimate of effect and is likely to change the estimate. Very low quality: we are very uncertain about the estimate. ^1^ The OR of study Silva 2011 is 1.07. ^2^ the OR of study Williams 2007a is 1.71. ^3^ The OR of study Silva 2011and Williams 2007a are 1.07, 1.71 respectively. ^4^ The OR of study Silva 2011and Williams 2007a are 1.07, 1.71 respectively, but the OR of Gawarammana 2004a is 0.92.

## Data Availability

The data supporting this META-ANALYSIS are from previously reported studies and data sets, which have been cited. The processed data are available in the supplementary files.

## Supplementary Materials

Supplementary Materials Appendix 1: Details of search strategy for PubMed. Appendix 2: The funnel plot of eight comparisons from four studies.
