# Supplementary figures and images for: Hydrocortisone for Preventing Adverse Drug Reactions to Snake Antivenom: A Meta-Analysis
Source: Emerg Med Int. 2022 Apr 22;2022:6151206. doi: 10.1155/2022/6151206 (PMC9054406; doi:10.1155/2022/6151206)

**Appendix 2** ：The funnel plot of eight comparisons from four studies


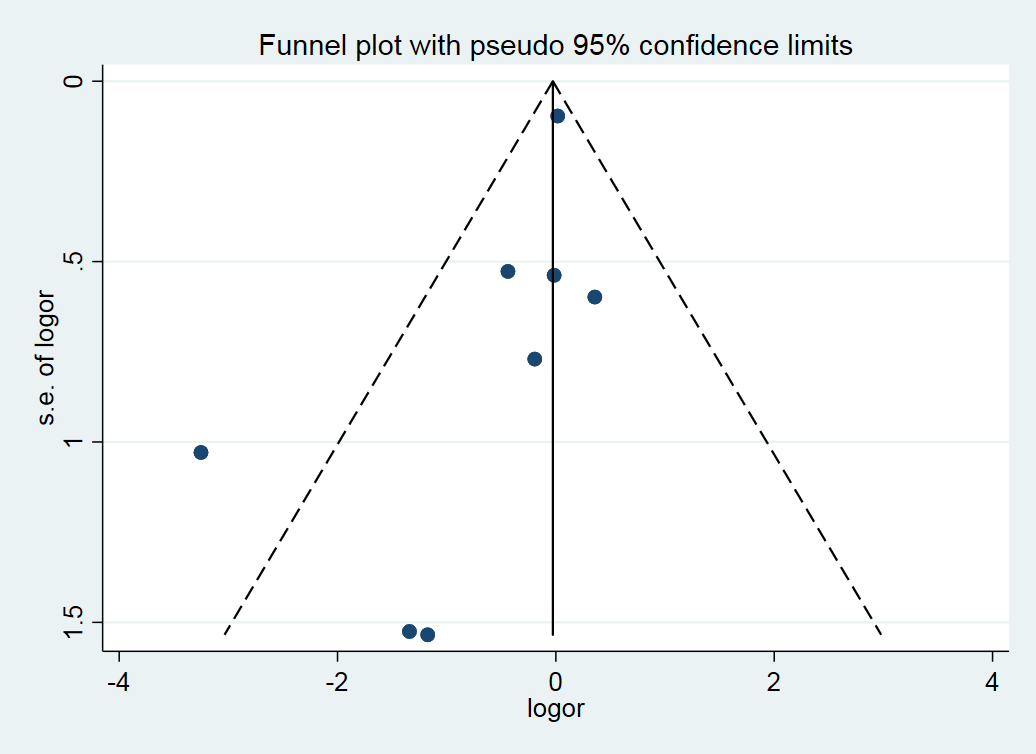

Supplement: Supplementary Materials — Appendix 1: Details of search strategy for PubMed. Appendix 2: The funnel plot of eight comparisons from four studies. [file 6151206.f1.zip › 6151206.f1/Appendix 2.docx]
