# Supplementary material for: Hydrocortisone for Preventing Adverse Drug Reactions to Snake Antivenom: A Meta-Analysis
Source: Emerg Med Int. 2022 Apr 22;2022:6151206. doi: 10.1155/2022/6151206 (PMC9054406; doi:10.1155/2022/6151206)
Supplement: Supplementary Materials — Appendix 1: Details of search strategy for PubMed. Appendix 2: The funnel plot of eight comparisons from four studies. [file 6151206.f1.zip › 6151206.f1/Appendix 1.doc]

**Appendix 1**

Details Search Strategy For Pubmed

1#: "Snake Bites"[Mesh]

2#: ((((((((((((((((((Snakebites) OR (Snake)) OR (Snakes)) OR (Snake Bite)) OR (Snakebite)) OR (Snake Envenomation)) OR (Envenomation)) OR (Envenomations)) OR (Snake Envenomations)) OR (Antivenoms)) OR (Anti-Venins)) OR (Anti Venins)) OR (Anti-Venoms)) OR (Anti Venoms)) OR (Antivenom)) OR (Anti-Venin)) OR (Anti Venin)) OR (Anti-Venom)) OR (Anti Venom)

3#: 1# OR 2#

4#": Anaphylaxis"[Mesh]

5#:((((((((Anaphylactoid) OR (Anaphylaxis)) OR (Anaphylactic)) OR (Reactions)) OR (Reaction)) OR (adverse reaction)) OR (adverse reactions)) OR (adverse effects)) OR (adverse effect)

6#:4# OR 5#

7#: "Steroids"[Mesh]

8#: "Glucocorticoids"[Mesh]

9#: (((((((((((((((((((((((((((((Steroid) OR (Glucocorticoid)) OR (Glucorticoid)) OR (hydrocortisone)) OR (Cortisol)) OR (11-Epicortisol)) OR (11 Epicortisol)) OR (Epicortisol)) OR (Cortifair)) OR (Cortril)) OR (dexamethasone)) OR (Methylfluorprednisolone)) OR (Hexadecadrol)) OR (Decameth)) OR (Decaspray)) OR (Dexasone)) OR (Dexpak)) OR (Maxidex)) OR (Millicorten)) OR (Oradexon)) OR (Decaject)) OR (Decaject-L.A.)) OR (Decaject L.A.)) OR (Hexadro)) OR (Methylprednisolone)) OR (Metipred)) OR (6-Methylprednisolone)) OR (6 Methylprednisolone)) OR (Urbason)) OR (Medrol)

10#:7# OR 8# OR 9#

11#: 3# and 6# and 9#
